# Low-Intensity Exercise and Pregnancy Outcomes: An Examination in the Nurses' Health Study II

**DOI:** 10.1089/whr.2021.0011

**Published:** 2021-09-15

**Authors:** SeonAe Yeo, Jae H. Kang

**Affiliations:** ^1^ School of Nursing, University of North Carolina, Chapel Hill, North Carolina, USA.; ^2^ Channing Division of Network Medicine, Brigham and Women's Hospital/Harvard Medical School, Boston, Massachusetts, USA.

**Keywords:** light exercise, obesity, physical activity, recreational activity, sedentary

## Abstract

***Background:*** The benefit of low-intensity exercise (LIE) during pregnancy is poorly understood at a time when few women participate in moderate or vigorous exercise. Using data from the Nurses' Health Study II (NHSII), we tested the hypothesis that women who engaged in more LIE before and during pregnancy experience fewer pregnancy complications.

***Methods:*** Among 116,429 U.S. female registered nurses (25–42 years of age) who were enrolled in NHSII in 1989, we included participants (36–50 years of age) who reported in 2001 or 2005 that they were pregnant and completed questionnaires about pregnancy “low-intensity exercise (yoga, stretching, toning),” and who in 2009, provided a full pregnancy outcome history. Multivariable-adjusted relative risk (RR) and 95% confidence intervals (CIs) were calculated between LIE and adverse pregnancy outcomes using log-binomial regression models.

***Results:*** Among 225 eligible pregnant participants, 71 (31.6%) reported engaging in any LIE. LIE was associated with lower preterm birth, but not significantly associated with pregnancy loss or other adverse pregnancy outcomes. The RR for any LIE for preterm birth was 0.31 (95% CI: 0.09–1.07), with a significant dose–response association [RR = 0.65 (95% CI: 0.48–0.89) per every 30-minute session]. Some suggestive inverse associations were also observed for other adverse pregnancy outcomes: the RR for any LIE for low birthweight was 0.35 (95% CI: 0.08–1.48); for preeclampsia/gestational hypertension was 0.51 (95% CI: 0.13–1.96); and for gestational diabetes was 0.64 (95% CI: 0.25–1.64).

***Conclusion:*** Pregnant women can include yoga, stretching, and toning exercise for promoting wellbeing.

## Background

More than 50% of pregnant women in the U.S. are obese, increasing their risk for serious complications, including preeclampsia, gestational hypertension (GHTN), and giving birth to preterm and/or low-birthweight infants.^1–4^ Physical activity (PA) is recommended to manage obesity; PA of moderate or vigorous intensity for at least 150 minutes each week greatly improves health for all, in all stages of life, including for women during pregnancy.^5,6^ Although being sedentary can lead to excess risk for maternal and child mortality and morbidity,^7^ most pregnant women reduce their PA, and only 8% meet the recommendations in the 3rd trimester.^8^ Behavioral approaches to increase moderate and vigorous PA in women late in pregnancy have been tested with limited success.^9^

In a small randomized controlled trial with sedentary overweight/obese pregnant women,^10^ investigators found that compared with walking (a moderate PA), to which pregnant women adhered poorly, 10 or more weeks of stretching exercise (a low PA) was much better adhered to with similar or better pregnancy outcomes.^11^ Another small randomized controlled trial (*n* = 68), which reported 1-hour yoga, three times a week in the second trimester, resulted in fewer cases of pregnancy-induced hypertension, preeclampsia, gestational diabetes (GDM), and intrauterine growth restriction than the usual care group.^12^ More recently, a quasi-experimental study (*n* = 100) reported that the group assigned to 1-hour yoga, three-times a week for 10 weeks reported better uterine artery function and fewer GDM and preeclampsia than the control group.^13^ Because of the paucity of data on this topic, more studies are needed.

The purpose of this study was to test the hypothesis that women who engaged in low-intensity exercise (LIE) before and during pregnancy experience fewer pregnancy complications. We used data from a sample extracted from a large cohort, where more than 100,000 women of reproductive age were followed. Investigators asked participants a wide range of questions about their health and lifestyle, including LIEs.

## Materials and Methods

### Study population

We used data from the Nurses' Health Study II (NHSII), an ongoing prospective study of 116,429 U.S. female registered nurses (25–42 years of age) who were enrolled in 1989. Participants complete self-administered questionnaires biennially on a variety of health, lifestyle, and reproductive characteristics; the cumulative follow-up of participants has been >85%. In 2001 and 2005, participants were asked about their engagement in low-intensity recreational PA as well as whether they were currently pregnant (in 2005, participants' ages ranged from 40 to 61 years, and few pregnancies were reported beyond 2005). In 2009, participants (45–62 years of age) reported on their life-time pregnancy history; thus, we could identify women who were currently pregnant as of 2001 or 2005 questionnaires and examine their self-reported pregnancy LIE as of 2001 or 2005, and prospectively evaluate pregnancy outcomes as self-reported in 2009.

Pregnant participants were asked in 2001 and 2005 about “low-intensity exercise (yoga, stretching, toning)” as part of a 10-item assessment of recreational activity. The question was “During the past year, what was your average time per week spent at each of the following recreational activities?.” Response options included 10 categories ranging from 0 minutes to ≥11 hours per week.

Among the original 116,429 participants, we excluded 10,764 women who did not complete questionnaires in either 2001 or 2005; 105,169 women who were not pregnant in both 2001 and 2005 questionnaire years; 111 women with missing PA information; and finally, 160 women who did not complete the biennial questionnaire in 2009 that collected comprehensive data on outcomes of lifetime pregnancies, or whose responses in 2009 could not be matched to pregnancies in 2001 or 2005 (*e.g*., they said in 2009 that they were pregnant in 2003). After exclusions, we had data from 225 eligible women, who were 36–50 years of age (median = 40 years) during their pregnancy. The NHSII protocol for the current study was approved by the Institutional Review Board of the Brigham and Women's Hospital and the Harvard T.H. Chan School of Public Health, Boston, MA, USA.

### Assessment of PA and Low LIEs

In 2001 and 2005, women reported the average time per week during the past year spent on 10 recreational activities: LIE (*e.g*., yoga, stretching, toning), walking, jogging, running, bicycling (including on a stationary machine), racquet sports (tennis, squash, racquetball), lap swimming, other aerobic activities (*e.g*., aerobic dance, ski or stair machine), weight training or resistance exercises (*e.g*., free weights or machines), and other high-intensity activities (*e.g*., lawn mowing). To incorporate the frequency, duration, and intensity of activity, we multiplied hours per week of each activity by its metabolic equivalent task score (MET). One MET equals 1 kcal/kg/hour, the energy expended by sitting quietly. We then summed values for all activities to create total MET hours/week.^14^ Among low-intensity activities, yoga and stretching were assigned as 2.5 METs^14^ and weekly MET-hours from LIE was calculated as: 2.5 MET × hours. For example, if a woman engaged in LIE for 30 minutes, three times a week or 1.5 hours, her total MET hours/week from LIE was 3.75 MET-hours/week. We calculated MET-hours/week for other activities and added MET-hours across all activities to determine women's total MET-hours/week.

In primary analyses, we determined whether a participant engaged in LIE and also treated LIE as a continuous variable with each unit increase representing a 1.25 MET-hour increase per week (equivalent to one 30-minute LIE session increase per week).

### Pregnancy outcomes

In 2009, participants were asked about their lifetime pregnancy history. Participants responded to questions about each pregnancy for the calendar year in which the pregnancy ended and its outcome (*e.g*., multiple gestation, spontaneous abortion (SAB), stillbirth, tubal/ectopic); duration of pregnancy in weeks; pregnancy complications (*e.g*., GDM, pregnancy-induced hypertension, preeclampsia); type of labor (spontaneous or induced labor) and birth (C-section or vaginal birth); and birthweight and gender [note: the question was phrased as “birth weight and gender” and response options were “boy” and “girl”; “sex” to represent biological label was not used in the questionnaire]. For our analysis, we evaluate these outcomes: SAB, preterm birth, low birthweight, C-section delivery, preeclampsia, GHTN, and GDM. Length of gestation in weeks was reported in 9 categories: <8, 8–11, 12–19, 20–27, 28–31, 32–36, 37–39, 40–42, 43+. SAB was defined as a fetal loss occurring at <20 weeks' gestation. Preterm births were those occurring at <37 weeks' gestation. GDM and preeclampsia/GHTN diagnoses were determined for those with pregnancies lasting 20 or more (20+) weeks consistent with diagnostic criteria. Birthweight in pounds for pregnancies lasting 20+ weeks was reported in 6 categories: <5, 5–5.4, 5.5–6.9, 7–8.4, 8.5–9.9, 10+. Low birthweight was defined as <5.5 lbs. Finally, for pregnancies lasting 20+ weeks, we analyzed data on type of labor and delivery: spontaneous versus induced labor, and C-section versus vaginal birth.

Validity of self-report adverse pregnancy outcome of this study cohort has been reported elsewhere.^15–18^ Briefly, 94% of GDM was confirmed; preeclampsia, 89%; preterm birth, 81% sensitivity; birthweight, 0.74 correlation. Sensitivity of pregnancy loss recall in the general population is estimated to be around 75%.^19,20^

### Covariate data

We obtained covariate data from the questionnaire of 2001 or 2005, in which a participant reported being pregnant or in 2009. For all analyses, we adjusted for *a priori* known^1^ factors associated with adverse pregnancy outcomes, including: age at reporting pregnancy (2001 or 2005), total number of prior pregnancies (assessed in 2009), body mass index [BMI (kg/m^2^) as of 2001 or 2005], moderate or vigorous PA (MET-hours/week as of 2001 or 2005), and multiple births (for only the outcomes of preeclampsia/GHTN, preterm birth assessed in 2009). We also attempted to adjust for three other important covariates, such as antidepressant use, race/ethnicity, and smoking, but because cases with these attributes were few (*n* < 3), when we added these variables, the regression model would not converge for five of six outcomes, so they were left out of models.

### Statistical analyses

Because the participant was the unit of analysis, if women reported pregnancies in both 2001 and 2005 (*n* = 3), we used data from the 2001 pregnancy only.

Relative risk (RR) and 95% confidence intervals (CI) were calculated between LIE and adverse pregnancy outcomes using log-binomial regression models.^21^ In a few instances, because models did not converge, we used log-Poisson regression, which provides consistent estimates of the RR when empirical standard errors are applied.^22^ The basic model was adjusted for age in years at pregnancy. Multivariable-adjusted models were additionally adjusted for BMI (during pregnancy in 2001 or 2005), moderate or vigorous MET-hours/week (during pregnancy in 2001 or 2005), and number of prior pregnancies. Analyses of preeclampsia/GHTN and preterm birth were additionally adjusted for multiple gestation (singleton, multiples). All tests were two-sided and α = 0.05. We used SAS Version 9.4 software (SAS Institute, Cary, NC) for all analyses.

## Results

Of the 225 eligible pregnant participants (186 identified in 2001; 39 in 2005), 71 (31.6%) reported engaging in LIE (yoga, stretching, toning) during pregnancy (“any LIE”) and 154 reported engaging in “no LIE.” Table 1 shows characteristics of these groups. Group values are age adjusted. On average, the LIE group had 2.0 MET-hours/week or ∼1 hour of LIE per week. The LIE group reported longer hours than the no LIE group for moderate/vigorous activity (MET-hours/week), indicating that during pregnancy, those who engaged in LIE also engaged in more moderate/vigorous physical activities than the no LIE group. Also, we found that 16.1% of the LIE group were categorized as obese [BMI ≥30 (kg/m^2^)], whereas 28.1 of the no LIE group were obese.

**Table 1.** Age-adjusted characteristics by engagement in any low-intensity exercise (LIE; yoga, stretching, toning) during pregnancy in the Nurses' Health Study II in 2001 or 2005

	Engaged in low-intensity exercise	
	No (*n* = 154)	Yes (*n* = 71)
Age (years; SD)^a^	39.8 (2.4)	40.7 (3.1)
Low-intensity physical activity (MET-hours/week) (SD)	0.0 (0.0)	2.0 (2.1)
Moderate/vigorous-intensity physical activity (MET-hours/week) (SD)	14.1 (17.5)	24.0 (23.4)
Total physical activity (MET-hours/week), mean (SD)	14.1 (17.5)	26.1 (24.0)
<10 MET-hours/week, %	55.3	31.1
Current BMI (kg/m^2^), mean (SD)	28.3 (6.0)	26.1 (5.8)
Obesity (BMI ≥30 kg/m^2^), %	28.1	16.0
Number of total pregnancies (SD)	3.8 (2.5)	3.5 (2.0)
Multiple gestation, %	4.2	1.0
Antidepressant use, %	0.1	0.1
Current smoking, %	1.2	4.9
Non-Hispanic White, %	96.9	99.4
Adverse pregnancy outcomes and complications		
Spontaneous abortion, %	5.9	8.3
Preterm, %	16.5	11.7
Low birthweight, %	13.1	11.3
C-section delivery, %	30.5	34.1
Pre-eclampsia, %	5.0	2.6
Gestational hypertension, %	6.4	6.5
Gestational diabetes, %	12.8	7.7

Values are means (SD) or percentages and are standardized to the age distribution of the study population.

^a^ Value is not age adjusted.

BMI, body mass index; MET-hours, metabolic equivalent task hours per week.

In analyzing the association between engagement in LIE and adverse pregnancy outcomes and complications, we found a significantly lower risk of preterm birth with greater LIE (Table 2). In multivariable-adjusted models, the RR for any LIE (group had mean of ∼1 hour/week of LIE) for preterm birth was 0.31 (95% CI: 0.09–1.07) and with every 30-minute session of LIE increase per week, we observed a significant 35% lower risk of preterm birth (RR = 0.65; 95% CI: 0.48–0.89). In addition to preterm birth, some suggestive inverse associations (not statistically significant) were also observed for other adverse pregnancy outcomes. In multivariable-adjusted models, the RR for any LIE for low birthweight was 0.35 (95% CI: 0.08–1.48); for preeclampsia/GHTN was 0.51 (95% CI: 0.13–1.96); and for GDM was 0.64 (95% CI: 0.25–1.64).

**Table 2.** Association (risk ratio and 95% confidence intervals) between engagement in any low-intensity exercise among pregnancies in the Nurses' Health Study II in 2001 or 2005 and adverse pregnancy outcomes and complications as reported in 2009

	Engaged in low-intensity exercise		Per 1.25 MET-hour (or one 30-minute session) increase in LIE/week
	No (*n* = 154)	Yes (*n* = 71)	
Spontaneous abortion^c^	Case/Total = 10/154	Case/Total = 6/71	
Age-adjusted RR^a^ (95% CI)	1.00 (ref)	1.25 (0.47–3.31)	
Multivariable-adjusted RR^b^ (95% CI)	1.00 (ref)	0.86 (0.33–2.20)	0.93 (0.70–1.24); p-linear trend = 0.62
Preterm birth^d^	Case/Total = 15/144	Case/Total = 4/65	
Age-adjusted RR^a^ (95% CI)	1.00 (ref)	0.54 (0.22–1.34)	
Multivariable-adjusted RR^b^ (95% CI)	1.00 (ref)	0.31 (0.09–1.07)	0.65 (0.48–0.89); p-linear trend = 0.01
Low birthweight^d^	Case/Total = 12/144	Case/Total = 2/65	
Age-adjusted RR^a^ (95% CI)	1.00 (ref)	0.35 (0.09–1.43)	
Multivariable-adjusted RR^b^ (95% CI)	1.00 (ref)	0.35 (0.08–1.48)	0.98 (0.65–1.47); p-linear trend = 0.91
C-section delivery^d^	Case/Total = 45/144	Case/Total = 22/65	
Age-adjusted RR^a^ (95% CI)	1.00 (ref)	1.04 (0.68–1.60)	
Multivariable-adjusted RR^b^ (95% CI)	1.00 (ref)	1.04 (0.66–1.62)	1.09 (0.97–1.22); p-linear trend = 0.15
Preeclampsia/Gestational hypertension^d^	Case/Total = 15/144	Case/Total = 3/65	
Age-adjusted RR^a^ (95% CI)	1.00 (ref)	0.45 (0.12–1.59)	
Multivariable-adjusted RR^b^ (95% CI)	1.00 (ref)	0.51 (0.13–1.96)	0.85 (0.48–1.52); p-linear trend = 0.59
Gestational diabetes^d^	Case/Total = 20/144	Case/Total = 5/65	
Age-adjusted RR^a^ (95% CI)	1.00 (ref)	0.54 (0.22–1.35)	
Multivariable-adjusted RR^b^ (95% CI)	1.00 (ref)	0.64 (0.25–1.64)	1.04 (0.79–1.38); p-linear trend = 0.83

^a^ Model adjusted for age only.

^b^ Multivariable-adjusted model additionally adjusted for current BMI (as of 2001 or 2005), number of total pregnancies, moderate or vigorous physical activity (MET-hours/week), multiple gestation (only for outcomes preeclampsia/GHTN and preterm birth).

^c^ Among pregnancies.

^d^ Among births.

RR, relative risk; CI, confidence interval.

LIE was not associated with pregnancy loss or C-section delivery. In multivariable-adjusted models, RRs of those who engaged in LIE, compared with those who did not, for SAB was 0.86 (95% CI: 0.33–2.20) and for C-section delivery was 1.04 (95% CI: 0.66–1.62).

## Discussion

We tested our hypothesis that women who engaged in LIE before and during pregnancy would experience better maternal–fetal outcomes within a sample from NHSII. We found that women experienced 49% less preeclampsia when women engaged in LIE preeclampsia affects between 2% and 8% of pregnancies worldwide.^23^ When pregnant women develop preeclampsia, the only known treatment is delivery of both the fetus and the placenta, often resulting in preterm birth of a growth-restricted neonate, which leads to deleterious consequences. Preeclampsia can result in cardiovascular complications for the mother as well as cardiometabolic disease to the offspring later in life.^24^ The etiology of preeclampsia remains unknown. A number of maternal risk factors have been recognized to identify high-risk pregnant women, including preconception obesity, chronic hypertension, and family history. It is hypothesized that proinflammatory cytokines found in adipose tissue cause aggravated systemic inflammatory responses and angiogenic imbalances in the circulation and in the placenta.^25^ A small study reported that LIE enhanced parasympathetic activity, which has a known calming effect.^26^ Thus, these various supportive lines of evidence may provide a framework to advance our scientific knowledge that may lead to clinical interventions to reduce preeclampsia.

We observed inverse associations between LIE during pregnancy on other outcomes, including GDM, preterm birth, and low birthweight. Women who engaged in LIE were 36% less likely (multivariable-adjusted RR: 0.54 [0.25, 1.64]) to have GDM. A small study reported a similar association with resistance exercise on the rate of GDM.^27^ The prevalence of GDM was significantly lower in the aerobic and resistant exercise group (2.1%; exercise involving skeletal muscle stretching by resistant band) compared with the aerobic exercise alone group (12.1%). A systematic review and meta-analysis study by Huang et al.,^28^ suggested that a potential mechanism underlying this association is that compared with the conventional treatment group, patients with GDM in the resistance exercise group had reduced the dosage of insulin needed, suggesting improved glucose metabolism due to resistance exercise.

Women who engaged in LIE were 69% less likely (multivariable-adjusted RR: 0.31 [0.09–1.07]) to have a preterm birth, with a significant dose–response association. Similar to this study, one study^29^ examined the association between yoga and preterm birth using a large cohort data (Australian Longitudinal Survey on Women's Health). In their sample of 1835 women, 304 women reported that they were pregnant and engaged in yoga regularly in 2009. The odds ratio of “premature birth” for those who engaged in yoga compared with those who did not was reported as 0.53 (95% CI: 0.22–1.28; *p* = 0.16).

Women who engaged in LIE were 65% less likely (multivariable-adjusted RR: 0.35 [0.08–1.48]) to have a low-birthweight baby. While preterm birth often results in low-birthweight infants, a few studies indicate potential mechanisms of protective effect against low birthweight. One suggestive mechanism involves the uterine and placental blood flow. Bouya et al.^13^ looked at the effect of yoga on the uterine and placental blood flow. It was hypothesized that yoga improves the uterine artery blood flow function. The investigators compared Doppler ultrasound indices of the uterine arteries and found that compared with the control group, yoga group demonstrated improvements in these indices, thus, concluding that yoga during pregnancy improves uterine blood flow function, thus resulting in increased birthweight. Stretching is a component of yoga, but an independent association with the uterine blood flow for stretching alone has not been reported. Given the scarce data, more studies are warranted.

Our study had several limitations. First, our study may not be broadly generalizable as our study population was a unique subset of NHS2 participants: the pregnancies were in mothers 36–50 years of age, whose age at first birth was much later than other participants, who were more likely to be of Asian or Hispanic descent and had healthier lifestyles (Supplementary Table S1). Furthermore, by design, eligible women had to provide pregnancy PA levels as well as be healthy enough to answer the 2009 questionnaire that was 4–10 years after their index pregnancy. Also, 97% of study participants were non-Hispanic white, so the generalizability of our findings to other racial/ethnic groups is limited, particularly as the CDC reported that African Americans suffer from higher preterm birth than other groups.^30^ Second, the validity of self-reported adverse pregnancy outcomes of the study cohort varied from 94% for GDM to 75% for pregnancy loss recall, which may have led to misclassification and biases toward the null. Also, there may have been other important factors (*e.g*., frequency and quality of prenatal care) that were not measured, so there might have been some residual confounding. We also acknowledge that because of the small sample size, we lacked adequate power to detect modest associations with several outcomes. However, the prospective cohort design was a strength of our study.

Although moderate/vigorous activities are important, LIE may possess its own spectrum of health effects. Unlike inactivity or sedentariness, which is strongly linked to increased mortality and morbidity, LIEs require large skeletal muscle contraction. Interestingly, in our study, women who engaged in more LIE also engaged in more moderate/vigorous activities; however, this was adjusted for, so the relation between LIE and pregnancy outcomes were statistically independent of moderate/vigorous activities. As such, effective exercise interventions may include both moderate- and low-intensity activities to improve pregnancy outcomes.

## Conclusions

In conclusion, we report preliminary evidence that LIE such as yoga, stretching, or toning, when practiced regularly by pregnant women, may be associated with a lower rate of preterm birth. Although health care providers continue to emphasize the importance of moderate-intensity exercise, such as brisk walking, pregnant women may also engage in LIEs.

## Ethics Approval and Consent to Participate

The NHSII protocol for the parent study was approved by the Institutional Review Board (IRB) of the Brigham and Women's Hospital and the Harvard T.H. Chan School of Public Health, Boston, MA, USA. This article was based on the secondary analysis of an existing limited dataset from the NHSII. The IRB of the University of North Carolina at Chapel Hill approved the data analysis study protocol, which did not require participants to consent.

## Availability of Data and Material

This study involved a secondary data analysis. The datasets used and/or analyzed during the current study can be accessed by submitting an online request. Further information, including the procedures to obtain and access data from the Nurses' Health Study II is described at www.nurseshealthstudy.org/researchers (contact email: nhsaccess@channing.harvard.edu)

## Supplementary Material

Supplemental data

## Abbreviations Used

BMI: body mass index
CI: confidence interval
GDM: gestational diabetes
GHTN: gestational hypertension
LIE: low-intensity exercise
MET: metabolic equivalent task
NHSII: Nurses' Health Study II
PA: physical activity
SAB: spontaneous abortion
