# Supplementary material for: Low-Intensity Exercise and Pregnancy Outcomes: An Examination in the Nurses' Health Study II
Source: Womens Health Rep (New Rochelle). 2021 Sep 15;2(1):389–95. doi: 10.1089/whr.2021.0011 (PMC8524727; doi:10.1089/whr.2021.0011)
Supplement: Supplemental data [file Suppl_TableS1.docx]

| **Supplemental Table 1.** Characteristics of Nurses’ Health Study II Participants by Inclusion into the LIE study | | |  |
| --- | --- | --- | --- |
|  | All gravid women as of 2009 (n=67063) | Participants included in the study (n=225) |  |
| Age in 1989, years | 34.6± 4.7 | 32.2± 2.9 |  |
| Age at first birth | 26.6± 4.8 | 38.2± 7.2 |  |
| Age at last birth | 30.8± 4.9 | 44.5± 2.7 |  |
| Total gravidity (number of pregnancies) | 2.8± 1.4 | 3.5± 1.7 |  |
| Total parity (number of live births) | 2.2± 1.1 | 2.5± 1.4 |  |
| Race: White, % | 96.6 | 89.3 |  |
| Black, % | 1.4 | 0.0 |  |
| Asian, % | 1.4 | 10.6 |  |
| Pacific Islander or American Indian, % | 0.6 | 0.1 |  |
| Ethnicity: Not Hispanic, % | 98.3 | 89.4 |  |
| Hispanic, % | 1.7 | 10.6 |  |
| Body Mass Index (kg/m^2^) in 1989 | 23.8± 4.7 | 22.5± 4.8 |  |
| Current smoker in 1989 % | 11.7 | 8.5 |  |
| Alcohol consumed per day in 1989, g/day | 2.8± 5.3 | 2.0± 2.5 |  |
| Marital status in 1989: Never , % | 6.8 | 36.1 |  |
| Married, % | 84.0 | 25.1 |  |
| Divorced, % | 7.3 | 22.3 |  |
| Separated or widowed, % | 1.7 | 16.5 |  |
| Values are means ± SD or percentages and are standardized to the age distribution of the study population. | | | |
